# Transcriptome Profiling and Molecular Therapeutic Advances in Cystic Fibrosis: Recent Insights

**DOI:** 10.3390/genes10030180

**Published:** 2019-02-26

**Authors:** Justin E. Ideozu, Xi Zhang, Susanna McColley, Hara Levy

**Affiliations:** 1 Ann & Robert H. Lurie Children’s Hospital of Chicago, Chicago, IL 60611, USA; justin.ideozu@northwestern.edu (J.E.I.); XiZhang@luriechildrens.org (X.Z.); SMcColley@luriechildrens.org (S.M.); 2 Human Molecular Genetics Program, Stanley Manne Children’s Research Institute, Chicago, IL 60614, USA; 3 Feinberg School of Medicine at Northwestern University Chicago, Chicago, IL 60611, USA

**Keywords:** cystic fibrosis, transcriptome profiling, molecular therapy, RNA-Seq, microarray

## Abstract

In cystic fibrosis (CF), mutations in the cystic fibrosis transmembrane conductance regulator (*CFTR*) gene disrupt the capacity of the encoded protein to function as a channel to transport chloride ions and water across cell membranes. The consequences are deleterious, system-wide, and immensely variable, even among patients with the same *CFTR* genotype. This underscores the need to characterize the mechanisms contributing to CF pathophysiology. Gene replacement and gene editing therapies have been pursued intensively and are expected to provide a one-time treatment for CF. However, gene replacement therapy is limited by the lack of efficient vectors to deliver functional copies of CFTR to cells without immunological complications, while gene editing technologies such as CRISPR/Cas9 are still in their infancy, mainly useful in somatic cells and limited by off-target insertions. Small molecule treatments targeted at potentiating or correcting CFTR have shown clinical benefits, but they are limited to a few *CFTR* mutations and insufficient to overcome challenges related to clinical heterogeneity. Transcriptome profiling approaches have emerged as robust tools capable of characterizing phenotypic variability and revealing novel molecular targets with therapeutic potential for CF. We summarize current insights gained through transcriptome profiling approaches in CF studies and recent advances in molecular therapeutics.

## 1. Introduction: Cystic Fibrosis

Cystic fibrosis (CF) is an autosomal recessive genetic disease due to mutations in the cystic fibrosis conductance regulator (*CFTR*) gene [1,2]. The gene encodes a member of the ATP-binding cassette (ABC) transporter superfamily that functions as a channel to transport chloride ions and water across cell membranes. Disruption of this crucial CFTR function results in deleterious system-wide consequences that vary immensely among CF patients [3]. This underscores the need to characterize molecular mechanisms driving CF pathophysiology.

**Figure 1:**
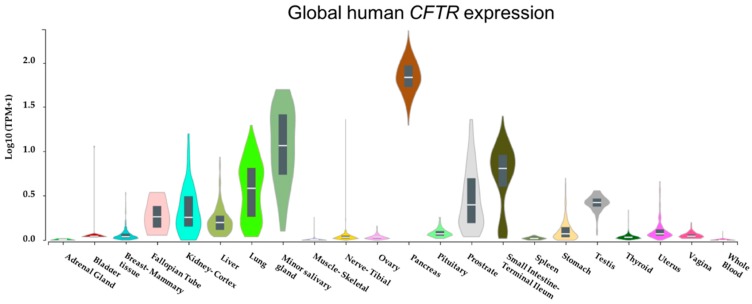
Cystic fibrosis transmembrane conductance regulator (*CFTR*) is abundantly expressed in many tissues. *CFTR* mRNA expression levels across several tissues were retrieved from Genotype-Tissue Expression (GTEx) Portal [5]. Expression values are shown in transcript per million (Log10) on the *y*-axis while *x*-axis represents tissues.

Although chronic progressive lung disease accounts for the vast majority of morbidity and mortality in CF, it is a multisystem disease affecting several body organs [4]. With our in silico analysis of global gene expression datasets from the Genotype-Tissue Expression (GTEx) project based on 53 non-diseased tissue sites from ~1000 individuals confirming *CFTR* is abundantly expressed in several tissues (Figure 1), it is conceivable that *CFTR* mutations may result in multisystem consequences.

To date, over 2000 *CFTR* mutations have been identified (http://www.genet.sickkids.on.ca/), with varying implications for the disease severity based on their interference with the structure and function of CFTR protein [4]. Gene replacement and gene editing therapies have been pursued intensively in search of a one-time cure for CF, but these have so far not led to convincing clinical results [6,7]. In contrast, small molecules targeted at correcting or potentiating CFTR have shown clinical benefits in recent times but there are concerns these therapies do not cover all known CFTR mutations [7], suggesting they are insufficient to resolve challenges met with clinical heterogeneity. With evidence from CF twin sibling studies [8] and genome-wide association studies (GWAS) [9] demonstrating that modifier genes contribute to phenotypic outcomes and variability in CF, there is a pressing need to better understand the underlying pathophysiological mechanisms of CF in order to identify novel molecular candidates that offer promise as predictors of prognosis or therapeutic targets.

Transcriptome profiling approaches have emerged as useful tools for delineating the transcriptome complexities in CF cells [10], in view of characterizing molecular mechanisms that contribute to the disease pathophysiology or identifying molecular targets with therapeutic potential. In this review, we provide current insights into the utilization of transcriptome profiling approaches in CF studies; we discuss prominent findings and advances of CF molecular therapies emphasizing on target identification via transcriptomics. We also discuss emerging areas for transcriptome profiling in CF and highlight some limitations of transcriptome profiling in CF. 

## 2. Transcriptome Profiling in Cystic Fibrosis

In attempts to better understand the complex sequence of transcriptional events influenced by dysfunctional CFTR, many CF studies have utilized high-throughput transcriptome profiling approaches (Table 1).

Transcriptome profiling provides a robust approach for simultaneous quantification of the expression signatures of all transcripts within a cell and to assess their expression differences across various physiological conditions [28]. When used in combination with in silico functional genomics approaches, complex mechanisms underlying the pathophysiology of many diseases can be deciphered [29,30,31]. Microarray and RNA-Sequencing are two powerful transcriptome profiling technologies that have proven resourceful for deducing and quantifying transcriptomes in many CF studies. While microarray array solutions are hybridization-based and focus on quantifying a predefined set of transcripts, RNA-Sequencing is sequence-based and offers an unbiased quantification of all transcripts within a cell without prior knowledge of a particular gene. In addition, RNA-Sequencing can provide information regarding alternative splicing and sequence variation in the captured genes [28,32]. A detailed review of transcriptome profiling approaches and advances in other human diseases are described elsewhere [28]. In CF, transcriptome profiling approaches are opening new horizons that will enhance our understanding of the disease pathology and unravel novel therapeutic targets (Table 1).

As shown in Table 1, microarray has been the most used transcriptome profiling approach in CF studies and most have investigated epithelial cells, which are the dominant cell-type affected by CFTR dysfunction in CF lung [33]. These studies have addressed questions ranging from identifying distinct genes that distinguish CF and its phenotypes to evaluating transcriptional responses of CF cells to external stimuli.

Alveolar macrophages (AM) were one of the earliest immune cells subjected to transcript profiling in CF. AM reside on the epithelial surface of the lung and play a crucial role in pathogen clearance [34], a process that is compromised in the CF lung [35,36]. Following stimulation of AM with early and late strains of CF *Pseudomonas aeruginosa* and *Burkholderia cepacia*, significant expression changes in genes involved in inflammatory pathways and signaling systems were observed, though similar responses were observed between early and late strains of both bacteria species [11]. This suggests that other host immune factors may be involved with the variable pulmonary phenotypes seen among CF patients.

As systemic markers have the potential to reflect the inflammation profile of the CF lung, blood cells have also emerged as targets for transcriptome profiling to delineate host immune factors associated with CF pathophysiology [17,19,26]. Transcriptional profiling has been direct, by assessing gene expression differences in CF blood cells [21,26] or indirect, by assessing transcriptional responses of blood cells exposed to CF-associated external stimuli [17,27], as previously described [37]. Although few studies have evaluated blood cells via transcriptomics in CF (Table 1), we have shown using microarray profiling that transcriptional signatures of peripheral blood mononuclear cells (PBMCs) exposed to plasma from patients with CF or from healthy controls (HC) can distinguish CF disease state from non-CF and characterizes its phenotypes. Like others (Table 1), we identified several genes that encode molecules within important biological pathways, especially those of the immune system are dysregulated in CF [17,27]. Others have also shown that transcriptional responses of PBMCs can enhance our understanding of immune dysfunction in CF. Following microarray profiling of CF PBMCs stimulated with flagellin, elevated levels of critical autophagosome marker LC3-II were observed. The elevated levels were effectively abolished by innate defense regulator (IDR)-1018, an anti-inflammatory peptide, which implicated dysfunctional autophagy for the exaggerated inflammatory responses seen in CF [19].

Further, a recent study utilizing transcriptome profiling by RNA-Sequencing of blood neutrophils identified 83 gene isoforms that demonstrated significant (False discovery rate-adjusted *p* < 0.05) changes in expression levels following treatment for exacerbations in CF [26]. Although functional enrichment was not performed, the results are indicative that alternative splicing events may be relevant to CF disease progression. Collectively, as shown in Table 1, findings from CF transcriptome profiling studies indicate that genes involved in many important biological pathways are dysregulated in CF.

## 3. Cystic Fibrosis-Associated Genes and Their Pathways

Pathway dysfunction is profound in CF and those involved in signal transduction and immune system are among the most documented in CF. Transcriptome profiling has identified key CF-associated genes relevant to the defective pathways (Table 2). We describe the prominent pathways here:

### 3.1. Signal Transduction Pathways

Signal transduction is essential for diverse cellular and molecular events including cell growth, proliferation, metabolism, and gene expression [38]. Several signaling pathways are defective in CF. For example, the PI3K/Akt/mTOR signaling pathway [39] is important for cell cycle regulation, with mTOR activation crucial in regulating cell autophagy [40]. Following mass spectrometry and functional analysis, it was found that mTORC1/2 and Eukaryotic Initiation Factor (EIF) signaling complexes interact with CFTR, mTOR is activated in CF bronchial epithelial cells, and the PI3K/Akt/mTOR pathway is inhibited in CF. Interestingly, the study also showed that inhibition of this pathway led to increased expression and stability of CFTR [39]. Thus, suggested the PI3K/Akt/mTOR pathway as a potential therapeutic target for CF. As another example, transcriptional profiling of CF PBMCs exposed to multiple bacterial ligands revealed that dysfunctional autophagy was implicated for heightened inflammatory responses and AMPK-Akt signaling was identified as a potential anti-inflammatory target [19]. We recently identified activated EIF2 signaling pathway as the most significant pathway associated with CF via microarray profiling using a plasma-induced PBMCs model [27]. Follow-up studies are needed to understand the mechanisms involved. Other signal transduction pathways associated with CF include Wnt/β-Catenin Signaling [41], G-protein coupled receptors (GPCR), and Ca^2+^ signaling pathway [1].

### 3.2. Immune System Pathways

Aberrant immune responses are prominent in CF. Dysfunctional CFTR leading to defective NF-κB signaling and neutrophilic inflammation is one of the most documented features of CF lung disease [42]. NF-κB mediates the expression of pro-inflammatory genes such as cytokines and chemokine, thereby playing a crucial role in regulating immune response [43]. CF fetal lungs were shown to have elevated NF-κB levels prior to pathogen exposure and increased activation of NF-κB regulated genes compared to the non-CF [44], implicating dysfunctional CFTR as the chief cause of the defective NF-κB signaling. Also, via gene expression profiling, several NF-κB proinflammatory genes were found to be over-expressed in CF cells in comparison to control cells. The activation of NF-κB was correlated with higher inhibitor of kappaB kinase (IKK) and activator protein-1 (AP-1) activity [13]. While these observations focused on epithelial cells, transcriptome profiling of AM induced with CF *P. aeruginosa* and with *B. cepacian* resulted in enriched NF-κB and cytokine signaling pathways [11]. Other dysregulated CF-associated immune pathways captured via transcriptome profiling of blood cells include altered immune response, B-and T-cell activation [17], cytokine signaling [11], and Type 1 interferon response [10] (Table 1).

## 4. Molecular Advances in Developing Therapeutics for Cystic Fibrosis

Over the past decade, commendable progress has been made in the search for novel molecular therapies for CF. Gene therapy by replacing or editing CF-associated molecules are at the forefront in this context. We summarize recent advances.

### 4.1. Gene Replacement Therapy and Gene Editing

Since the discovery of the *CFTR* gene in 1989, gene replacement therapy has been pursued extensively as an option to replace mutated *CFTR* in CF cells with functional copies of the gene. The approach is promising as a one-time treatment for people with CF, but thus far, it has not led to convincing clinical results [6]. A major barrier to gene replacement therapy is the development of efficient vectors that enable safe delivery of functional *CFTR* to CF cells without immunological complications [7]. Addressing the barriers to gene transfer is expected to lead to the generation of effective molecular therapy for CF [7]. In contrast to gene replacement, gene editing approaches are aimed at using nucleases to enzymatically correct mutated genes in their native location in a cell. Several gene editing tools exist but one of the most revolutionary gene editing tools that hold promise for correcting mutated *CFTR* and restoring its function is CRISPR/Cas9 [45]. The technology uses a protein-RNA complex composed of Cas9 enzyme that binds to a guide RNA (gRNA) molecule to recognize the targeted DNA sequences. When utilized to repair *CFTR* locus in human intestinal stem cells, the technology robustly restored CFTR function [46]. Although the technology also faces barriers such as off-target insertion [47], it remains at the forefront in the search for a one-time cure for CF. Detailed review of recent advances in CF gene therapy are described elsewhere [7].

### 4.2. Cystic Fibrosis-Associated Molecules: Small and Large Molecular Targets for Treating Cystic Fibrosis

Although gene therapy has not yielded convincing clinical benefits, progress in therapeutics to relieve CF symptoms has been based on an increased understanding of the basic CFTR defects and the identification of novel molecular markers using advanced genetic technologies. Generally, mutations fall into six classes based on the mechanism by which they disrupt the structure and function of CFTR. The dysfunction may be due to the lack of CFTR protein synthesis (Class 1), failure of CFTR to reach cell surface due to misfolding and degradation (Class 2), reduced CFTR channel opening due to defects in gating (Class 3), reduced chloride conductance due channel defects (Class 4), reduced CFTR synthesis due to abnormal splicing (Class 5), and reduced CFTR stability at the cell surface (Class 6) [6].

Small molecule pharmacological agents developed to target basic CFTR defects have already yielded remarkable results in clinical trials (Table 3). These agents are termed CFTR modulators and include CFTR potentiators, correctors, and amplifiers. Potentiator therapies such as Ivacaftor increase flow of chloride ions by activating the CFTR channels. Thus, are helpful for CF patients with gating defects (Class 3) [48]. Correctors such as lumacaftor and tezacaftor facilitate proper maturation and delivery of CFTR protein to the cell surface. Patients with Class 2 CFTR mutation are the primary targets for correctors.

As both transport of chloride ions and delivery of CFTR protein to the cell surface are equally important to relieve the symptoms of CF, CFTR combination therapies using lumacaftor/ivacaftor or tezacaftor/ivacaftor have emerged effective agents for treating patients with Class 2 [45]. Amplifiers such as PTI-428 facilitate CFTR protein synthesis and are currently being tested in clinical trials (Table 3).

Beyond CFTR modulators, several other small molecule agents targeting other CF-associated genes have already completed phase 4 in clinical trials. For example, previous studies showing that increased levels of Matrix metallopeptidase (MMP-9) is associated with more severe lung disease in CF prompted the development of doxycycline, an antibiotic that is also a potent inhibitor for MMP-9, as a CF therapeutic [50]. In addition, with studies showing that CF patients lack sufficient levels of vitamin D crucial for host defense against microorganisms, Cholecalciferol was developed as an agonist targeting the vitamin D receptor (*VDR*) gene which encodes the nuclear hormone receptor for vitamin D3 [51] (Table 3).

Although small molecule agents have produced tremendous clinical results, large molecule agents are becoming increasingly important candidates for CF therapies. Large protein molecules or biologics are copies of endogenous human proteins that act by binding to specific cell receptors associated with a disease process [52]. Several drugs targeting genes encoding receptor molecules are currently being tested in clinical trials for CF. These include growth hormone receptor (*GHR*), interferon-γ receptor 1 (*IFNGR1*), interferon-γ receptor 2 (*IFNGR2*), and insulin receptor (*INSR*) (Table 3). Although these four targets were initially identified with other screening methods to be dysregulated in CF [49], *IFNGR1*, and *IFNGR2* were among the top genes we identified via transcriptome profiling of CF plasma-induced signatures [27]. Thus, the findings are an indication that advanced high-throughput transcriptome profiling methods hold promise as robust tools for identification of novel molecular targets with therapeutic potential in CF.

## 5. Emerging Targets for Transcriptome Profiling

As shown in Table 1, the vast majority of CF studies have focused on differential gene expression analysis. We describe emerging targets for transcriptome profiling below.

### 5.1. Non-Coding RNAs

Expression profiling of non-coding RNAs (ncRNAs) is emerging as an approach for identifying new therapeutic targets for CF, based on the crucial role these molecules play as master regulators of gene expression and biological processes [53]. Although there are several species of ncRNAs, microRNAs (miRNAs) and long non-coding RNAs (lncRNAs) are the two most studied regulatory ncRNAs because they have been implicated in many human diseases [54].

Briefly, miRNAs are short single-stranded small ncRNA species (20–25 nucleotides in length) that modulate the expression of their target genes by base-pairing with their complementary sequence. The altered expression of several miRNAs has been reported in a wide range of human diseases including cancers [55,56] and chronic respiratory diseases such as asthma, chronic obstructive pulmonary disease, and CF [57]. In CF, transcriptome profiling has provided some insights into their abundance in epithelial cells and their potential role in the disease progression [54,58,59]. For example, increased expression levels of miR-155 was observed in CF bronchial epithelial cells via microarray profiling which implicated proinflammatory expression of IL-8 [59]. In addition, expression profiling via microarray identified 92 miRNAs differentially expressed between CF and non-CF bronchial brushings. In particular, altered expression of miR-126 was associated with impaired innate immune responses in the CF airway [60]. Because these miRNAs target important genes involved in many immune pathways, their altered expression is highlighted to have deleterious consequences for innate immune responses triggered in the CF airway [54].

Similar to miRNAs, lncRNAs can act as activators or repressors of gene expression. LncRNAs are large (>200 nucleotides in length) ncRNA species that localize to the nucleus or cytoplasm, with a capacity to interact with DNA, RNA, and protein [61]. The regulatory role of lncRNAs in biological processes such as chromatin modification, transcription, and post-transcriptional processing, and intracellular trafficking is well-documented [53,62]. Aberrant expression of lncRNAs has been implicated in many respiratory diseases [63] including CF, where BGas lncRNA was demonstrated to regulate *CFTR* expression [64]. Although the identification and functional characterization of other lncRNAs potentially relevant to CF have not been pursued extensively, transcriptome profiling approaches are beginning to provide new insights into their role in CF. Following microarray profiling of 30,586 lncRNAs, a total of 1063 lncRNAs from bronchial epithelial cells were identified as differentially expressed between CF and non-CF samples. The aberrant expression of these lncRNAs were suggested to play crucial roles in CF lung infection and inflammation [65]. More recently, by analyzing the transcriptome datasets from primary CF and non-CF epithelial cells infected with *P. aeruginosa*, investigators identified a unique lncRNA profile characterizing CF and potentially relevant to the impaired immune response seen in patients [66]. In addition, a recent study utilizing transcriptome profiling by microarray identified 91 ncRNAs dysregulated in CF airway epithelial cells. Further mechanistic analyses indicated linc-SUMF1-2 was associated with the aberrant expression of genes such as MYC and CXCL10 in CF airway epithelial cells [67].

As the expression of ncRNAs can be modulated in vitro or in vivo to mediate the expression of their target gene [68], including *CFTR* [69], the identification of dysregulated ncRNAs in CF cells may open new horizons for therapeutics.

### 5.2. Alternate Splicing and Cystic Fibrosis-Relevant Transcript Isoforms

Alternative splicing is an important biological mechanism through which most mammalian genes generate distinct transcript isoforms that can have diverse functions in different cell-types or disease states [70,71]. Mammalian splicing machinery is regulated by splicing factors that encode protein molecules that facilitate the precise selection of splicing sites and subsequent splicing processes essential for mRNA maturation and protein synthesis [72]. Disruption of these splicing programs can negatively impact on cellular functions and potentially result in human diseases [71]. Both altered expression levels of splicing factors and transcript isoforms can be simultaneously quantified via transcriptome profiling methods. This approach has already led to the discovery and characterization of several dysregulated disease-specific isoforms in recent times [71,73,74]. Although the literature is still sparse with regard to genome-wide implications of aberrant alternative splicing in CF progression, a recent study utilizing transcriptome profiling by RNA-Sequencing identified 83 transcript isoforms in blood neutrophils that distinguished CF following treatment for exacerbation [26]. Thus, identifying the CF-specific isoforms of key genes may be useful for discovering novel diagnostic and therapeutic targets.

## 6. Challenges for Cystic Fibrosis Transcriptomics Studies

Although transcriptome profiling holds promise for revolutionizing our understanding of the molecular complexities of CF and its phenotypes in any cell type, barriers to this approach include the lack of useful model systems and cellular heterogeneity. These challenges limit the capacity of these advanced technologies to identify new therapeutic opportunities in CF.

### 6.1. Lack of Useful Model Systems

The availability of well-characterized model systems for transcriptome profiling has been instrumental in understanding the pathogenesis of many diseases and developing effective therapies [75]. Shortly after the discovery of the *CFTR* gene in 1989, the first mouse models were developed [76,77]. These models enhanced our understanding of basic CFTR function but failed to exhibit the characteristic lung and pancreatic phenotypes seen in humans [77]. A few years later, there were calls for utilizing other animal models that share close CFTR protein sequence similarity with humans [78]. The CFTR-Knockout pig and ferret exhibiting human phenotypic characteristics were then generated about a decade ago [79,80]. Much knowledge about the major consequences of dysfunctional CFTR such as impaired immune responses has been enhanced by intensively studying animal models and cell lines [80]. Impressively, these models also led to the development of therapies that have successfully improved health outcomes in patients [48]. However, with over 2000 *CFTR* mutations identified so far that can result in phenotypic differences [4], there is a pressing need to identify useful model systems that can effectively characterize variability in CF phenotypes and allow us to take advantage of advanced high-throughput transcriptomic methods.

One human model system that can be used effectively to distinguish disease phenotypes at transcriptome level is the human PBMCs-based model. This system reflects host immune cell responses to external stimuli that can be measured quantitatively at mRNA level using transcriptome profiling approaches. Human PBMCs stimulated with plasma or serum from patients followed by transcriptome profiling have provided new insights into diseases [81,82]. Recently, we used this model to reveal dysregulated expression of a diverse range of genes encoding molecules crucial for important immune pathways in CF [17,27]. Although this model system is limited by the cellular heterogeneity of PBMCs, it provides a useful resource to quantitatively profile immune cells via transcriptomic approaches. Of note, recent advances in gene editing technologies such as CRISPR/Cas9 are expected to generate new CF models that will eventually overcome the challenge due to the lack of model systems [80].

### 6.2. Bulk Transcriptome Profiling

Although transcriptome profiling approaches have fueled many important discoveries in CF (Table 1), most studies have performed bulk transcriptome profiling, which implies that the generated gene expression data may not be driven by individual cells but by the thousands to millions of cells present in the sample. As different cell-types within the same population can have different transcriptional profiles, which may also be influenced by different physiological conditions and disease states [83], it has become imperative to decipher cellular heterogeneity at a single-cell level.

New computational algorithms and transcriptome profiling at single-cell resolution have emerged as novel approaches to tackle challenges due to bulk sampling. Several computational methods capable of delineating cellular heterogeneity based on gene expression datasets generated from bulk transcriptome profiling have been developed [84,85,86]. However, a major drawback of the computational approaches is their inability to profile and distinguish the transcriptome of individual cell-types present in the sample. Single cell transcriptome profiling by RNA-Sequencing method (scRNA-Seq) have recently emerged as a robust approach to simultaneously identify novel cell-types, estimate cell-type abundance, and quantify gene expression levels at a single-cell resolution [83,87]. Although scRNA-Seq has not been utilized to extensively characterize CF and its phenotypes, a recently published study employing scRNA-Seq to characterize human and mouse airway epithelial cells identified pulmonary ionocyte as a new CFTR-enriched cell-type [87]. Generally, scRNA-Seq is still at an early stage for use in transcriptome profiling and are very expensive and computationally very demanding. However, it offers an apparent solution to the challenges posed by bulk transcriptome profiling and may open new avenues for CF therapy.

## 7. Conclusions and Future Directions

In just a few years, transcriptome profiling has expanded our understanding of CF pathophysiology. We have learned from the past studies that mutations in *CFTR* gene result in aberrant expression of genes encoding molecules relevant to several altered biological pathways in CF, prominently signal transduction and immune pathways. Although much of this knowledge seems to have been gained by studying airway epithelial cells, few studies have analyzed blood cells. Together, these studies indicate impaired immune responses is a dominant feature of CF. With data from GTEx confirming that *CFTR* is abundantly expressed in many unexplored tissues and organs, future studies focusing on such targets will elucidate molecular mechanisms underlying how dysfunctional *CFTR* results in a multisystem CF disease. Beyond the identification of dysregulated genes in CF cells, identification of CF-specific isoforms from multiple-isoform genes may reveal novel molecular targets for specific therapy. Current knowledge of dysregulated genes in CF transcriptomes are based on analyzing bulk cells. Recent computational algorithms and novel molecular methods such as scRNA-Seq will allow researchers to decipher cellular heterogeneity in gene transcription signatures. Taking advantage of transcriptome profiling will open new horizons for better and specific therapies for CF.

## Figures and Tables

**Table 1:** Summary of human transcriptome profiling studies in cystic fibrosis.

Year	Focus	Methods	Tissue/Cell	Key Enriched Pathways	Reference
**2005**	Transcriptional changes induced by CF *Pseudomonas aeruginosa* and *Burkholderia cepacia*	Microarray	Alveolar macrophages	Cytokine signaling; NF-κB signaling	[11]
**2006**	Mild vs. severe CF lung disease	Microarray	Epithelial	Ubiquitin cycle; lipid metabolism	[12]
**2007**	CF vs. healthy controls	Microarray	Epithelial	Activator protein 1 and NF-κB activator pathway	[13]
**2009**	Transcriptional changes induced by Azithromycin	Microarray	Epithelial	Lipid/cholesterol biosynthesis; cell division	[14]
**2011**	CF vs. non-CF samples	Microarray	Epithelial	Inflammatory response; cell-to-cell signaling; cellular movement	[15]
**2012**	Transcriptional changes induced by CF *P. aeruginosa*	Microarray	Epithelial	TLR signaling; chemokine signaling	[16]
**2012**	Transcriptional changes induced by plasma of CF and non-CF	Microarray	PBMCs	Immune response, B- and T-cell activation	[17]
**2013**	CF vs. healthy controls	Microarray	Epithelial	Inflammation; defense response	[18]
**2013**	Transcriptional changes induced by innate defense regulator 1018	Microarray	Epithelial, PBMCs	Dysfunctional autophagy, AMPK-Akt signaling	[19]
**2013**	Transcriptional changes influenced by a CF modifier gene-*EHF*	Microarray	Epithelial	Gene regulation; glycosylation of biopolymers	[20]
**2014**	Transcriptional changes influenced by CF and other lung diseases	Microarray	Blood, PBMCs	Immune response, leukocyte activation in immune response	[21]
**2014**	Transcriptional changes induced by oxidative stress	Microarray	Epithelia	Cell survival; regulation of signal transduction	[22]
**2015**	Transcriptional changes influenced by CF	Microarray	Lymphoblasts	Endomembrane function; ER response to stress	[23]
**2017**	Mild vs. severe lung phenotype	RNA-Seq	Leukocytes	Type 1 interferon response; protein targeting ER	[10]
**2017**	Transcriptional changes induced by digitoxin	Microarray	Epithelial	Inflammatory pathway; immune response	[24]
**2018**	Transcriptional changes influenced by genomic variation	RNA-Seq	Epithelial	Inflammation/inflammatory signaling; innate immune response	[25]
**2018**	CF before vs. after treatment for exacerbation	RNA-Seq	Blood, neutrophils	Functional enrichment not performed; inflammasome genes	[26]
**2018**	Transcriptional changes induced by plasma of CF and its phenotypes	Microarray	PBMCs	E1F2 Signaling, IL-8 signaling, B-cell receptor, production of nitric oxide and oxygen reactive species	[27]

CF, cystic fibrosis; NF-κB, nuclear factor kappa B; TLR, Toll-like receptor; AMPK, AMP-activated protein kinase; ER, endoplasmic reticulum; IL, interleukin; PBMC, peripheral blood mononuclear cell.

**Table 2:** Altered pathways in CF and associated genes.

Gene	Gene Name	Relevant Pathway(s)	References
Signal Transduction/Transport of Small Molecules			
*CDH4*	Cadherin 4	Wnt signaling	[13]
*CDH8*	Cadherin 8	Wnt signaling	[13]
*CDK6*	Cyclin dependent kinase 6	PI3K/Akt signaling	[13,27]
*CHRM3*	Cholinergic receptor muscarinic 3	Signaling by GPCR, Calcium signaling pathway	[22,27]
*ESR1*	Estrogen receptor 1	Signaling by GPCR	[22,27]
*FGF2*	Fibroblast growth factor 2	mTOR signaling, PI3K/Akt signaling	[13,27]
*ITGA4*	Integrin subunit α 4	PI3K/Akt signaling	[13]
*ITGA6*	Integrin subunit α 6	Signaling by GPCR, Wnt signaling	[13,27]
*JAG1*	Jagged 1	PI3K/Akt signaling, Signaling by GPCR	[13,27]
*MMP9*	Matrix metallopeptidase 9	Signaling by GPCR, Interleukin 4 & 13 signaling	[22]
*NCF2*	Neutrophil cytosolic factor 2	Signaling by GPCR	[22,27]
*NOTCH3*	Notch 3	PI3K/Akt signaling, Signaling by GPCR	[13,27]
*NRP2*	Neuropilin 2	Signaling by GPCR	[13,22,27]
*PIK3R1*	Phosphoinositide-3-kinase regulatory subunit 1	mTOR signaling, PI3K/Akt signaling	[22,27]
*PSMA5*	Proteasome subunit α 5	Signaling by GPCR, Wnt signaling	[22,27]
*SFN*	Stratifin	mTOR signaling, Signaling by GPCR	[13,27]
*SFRP1*	Secreted frizzled related protein 1	Signaling by GPCR, Wnt signaling	[22,27]
*SOX9*	SRY-box 9	Signaling by GPCR, Wnt signaling	[13,22,27]
*STAT1*	Signal transducer and activator of transcription 1	PI3K/Akt signaling, Signaling by GPCR	[22,27]
*WNT2B*	Wnt family member 2B	Wnt/β-catenin signaling, PI3K/Akt signaling	[13,27]
**Immune system**			
*CTSB*	Cathepsin B	Innate immune system, Bacterial infections in CF airways	[13]
*CXCL1*	C-X-C motif chemokine ligand 1	Cytokines and inflammatory response, immune response	[13,22,27]
*CXCL10*	C-X-C motif chemokine ligand 10	Cytokine signaling, Signaling by interleukins	[13,22,27]
*CXCL8*	C-X-C motif chemokine ligand 8	NFkB signaling, Innate immune system, Interleukin signaling	[22,27]
*GHR*	Growth hormone receptor	Cytokine signaling, innate immune system	[22]
*HMOX1*	Heme oxygenase 1	NFkB signaling, Cytokine signaling; Innate immune system	[13,22]
*ICAM1*	Intercellular adhesion molecule 1	Cytokine signaling, Signaling by interleukins	[13,27]
*IL1A*	Interleukin 1 α	NFkB, T-cell receptor signaling, IFN α & β immune response	[13,22,27]
*IL1B*	Interleukin 1 β	Cytokine signaling, immune response, Lung fibrosis	[13,22,27]
*IL6*	Interleukin 6	Cytokine signaling, immune response	[13,22,27]
*ITGA4*	Integrin subunit α 4	Transcriptional regulation by RUNX3, Generic transcription	[13]
*LY96*	Lymphocyte antigen 96	NFkB signaling, Innate immune system	[13,27]
*MME*	Membrane metalloendopeptidase	B-cell development pathways, innate immune system	[13]
*MMP1*	Matrix metallopeptidase 1	Cytokine signaling, immune response	[13]
*NOX4*	NADPH oxidase 4	Immune cell transmigration, PAK pathway	[13]
*PTGS2*	Prostaglandin-endoperoxide synthase 2	NFkB signaling, Cytokine signaling; Innate immune system	[13]
*RASGRP1*	RAS guanyl releasing protein 1	T-cell receptor signaling; Cytokine signaling	[13,22,27]
*SERPINA1*	Serpin family A member 1	Innate immune system, Lung fibrosis	[13,22,27]
*TLR4*	Toll like receptor 4	NFkB signaling, Innate immune system, TREM1 signaling	[13,27]
*VEGFA*	Vascular endothelial growth factor A	Innate immune system, Signaling by interleukins	[13]

CF, cystic fibrosis; GPCR, G-protein coupled receptor; IL, interleukin; PI3K, phosphoinositide 3-kinase; NF-κB, nuclear factor kappa B; TREM, Triggering Receptor Expressed on Myeloid Cells; RUNX3, Runt Related Transcription Factor 3; PAK, p21-activated protein kinase; IFN, interferon.

**Table 3:** Gene targets and therapeutic advances.

Drug Name	Phase	Type	Status	Activity	Target	Target Class
Ivacaftor, lumacaftor	4	SM	Completed	Agonist	*CFTR*	Cystic fibrosis transmembrane conductance regulator (CFTR)
Doxycycline	4	SM	Completed	Antagonist	*MMP7*	Metallo protease M10A subfamily
Doxycycline	4	SM	Completed	Antagonist	*MMP1*	Metallo protease M10A subfamily
Cholecalciferol	4	SM	Completed	Agonist	*VDR*	Nuclear hormone receptor subfamily 1 group I member 1
Doxycycline	4	SM	Completed	Antagonist	*MMP13*	Metallo protease M10A subfamily
Doxycycline	4	SM	Completed	Antagonist	*MMP8*	Metallo protease M10A subfamily
Somatropin	3	Protein	Completed	Agonist	*GHR*	Membrane receptor
Denufosol	3	SM	Completed	Agonist	*P2RY2*	Purine receptor
Insulin glargine	3	Protein	Completed	Agonist	*INSR*	Tyrosine protein kinase InsR family
Prednisone	3	SM	Recruiting	Agonist	*NR3C1*	Nuclear hormone receptor subfamily 3 group C member 1
Tiotropium	3	SM	Completed	Antagonist	*CHRM3*	Acetylcholine receptor
Nitric oxide	2	SM	Recruiting	Agonist	*GUCY1A2*	Soluble guanylate cyclase
PTI-428	2	SM	Completed	Agonist	*CFTR*	CFTR
pGM169/GL67A	2	SM	Completed	Agonist	*CFTR*	CFTR
Digitoxin	2	SM	Completed	Antagonist	*ATP1B3*	Hydrolase
Nitric oxide	2	SM	Completed	Agonist	*GUCY1B1*	Soluble guanylate cyclase
Nitric oxide	2	SM	Completed	Agonist	*GUCY1B2*	Soluble guanylate cyclase
Nitric oxide	2	SM	Completed	Agonist	*GUCY1A1*	Soluble guanylate cyclase
Gallium nitrate	2	SM	Completed	Antagonist	*RRM2*	Enzyme
Sildenafil	2	SM	Active	Antagonist	*PDE5A*	Phosphodiesterase 5A
Digitoxin	2	SM	Completed	Antagonist	*ATP1A1*	Hydrolase
Omeprazole	2	SM	Recruiting	Antagonist	*ATP4B*	Hydrogen potassium ATPase
Miglustat	2	SM	Completed	Antagonist	*UGCG*	Transferase
P-1037	2	SM	Completed	Antagonist	*SCNN1B*	Epithelial sodium channel
Gallium nitrate	2	SM	Completed	Antagonist	*RRM2B*	Enzyme
Fiboflapon	2	SM	Completed	Antagonist	*ALOX5AP*	Other cytosolic protein
Losartan	2	SM	Recruiting	Antagonist	*AGTR1*	Angiotensin receptor
Digitoxin	2	SM	Completed	Antagonist	*FXYD2*	Hydrolase
Digitoxin	2	SM	Completed	Antagonist	*ATP1A2*	Hydrolase
Digitoxin	2	SM	Completed	Antagonist	*ATP1B2*	Hydrolase
Amiloride	2	SM	Completed	Antagonist	*SCNN1A*	Epithelial sodium channel
P-1037	2	SM	Completed	Antagonist	*SCNN1G*	Epithelial sodium channel
Digitoxin	2	SM	Completed	Antagonist	*ATP1A4*	Hydrolase
Digitoxin	2	SM	Completed	Antagonist	*ATP1A3*	Hydrolase
Digitoxin	2	SM	Completed	Antagonist	*ATP1B1*	Hydrolase
Gallium nitrate	2	SM	Completed	Antagonist	*RRM1*	Enzyme
Simvastatin	1	SM	Completed	Antagonist	*HMGCR*	Oxidoreductase
Hydroxychloroquine	1	SM	Completed	Antagonist	*TLR9*	Toll-like and Il-1 receptors
Amelubant	1	SM	Completed	Antagonist	*LTB4R*	Leukotriene receptor
Pioglitazone	1	SM	Completed	Agonist	*PPARG*	Nuclear hormone receptor subfamily 1 group C member 3
Interferon γ-1b	1	Protein	Completed	Agonist	*IFNGR2*	Membrane receptor
Hydroxychloroquine	1	SM	Completed	Antagonist	*TLR7*	Toll-like and Il-1 receptors
Interferon γ-1b	1	Protein	Completed	Agonist	*IFNGR1*	Membrane receptor

SM: Small molecule; InsR, insulin receptor; IL, interleukin. Source: Open target platform [49].
